# Patient perspectives of managing fatigue in Ankylosing Spondylitis, and views on potential interventions: a qualitative study

**DOI:** 10.1186/1471-2474-14-163

**Published:** 2013-05-09

**Authors:** Helen Davies, Sinead Brophy, Michael Dennis, Roxanne Cooksey, Elizabeth Irvine, Stefan Siebert

**Affiliations:** 1 College of Medicine, Swansea University, Swansea, South Wales, UK; 2 Institute of Infection Immunity and Inflammation, University of Glasgow, Glasgow, Scotland, UK

## Abstract

**Background:**

Fatigue is a major component of living with ankylosing spondylitis (AS), though it has been largely over-looked, and currently there are no specific agreed management strategies.

**Methods:**

This qualitative exploratory study involved participants who are members of an existing population-based ankylosing spondylitis (PAS) cohort. Participants residing in South West Wales were invited to participate in a focus group to discuss; (1) effects of fatigue, (2) self-management strategies and (3) potential future interventions. The focus groups were audio-recorded and the transcripts were analysed using thematic analysis.

**Results:**

Participants consisted of 3 males/4 females (group 1) and 4 males/3 females (group 2), aged between 35 and 73 years (mean age 53 years). Three main themes were identified: (1) The effects of fatigue were multi-dimensional with participants expressing feelings of being *‘drained’* (physical), *‘upset’* (emotional) and experiencing *‘low-mood’* (psychological); (2) The most commonly reported self-management strategy for fatigue was a balanced combination of activity (exercise) and rest. Medication was reluctantly taken due to side-effects and worries over dependency; (3) Participants expressed a preference for psychological therapies rather than pharmacological for managing fatigue. Information on Mindfulness-Based Stress Reduction (MBSR) was received with interest, with recommendations for delivery in a group format with the option of distance-based delivery for people who were not able to attend a group course.

**Conclusions:**

Patients frequently try and manage their fatigue without any formal guidance or support. Our research indicates there is a need for future research to focus on psychological interventions to address the multi-faceted aspects of fatigue in AS.

## Background

Fatigue affects those in poor and good health and is a multi-determined phenomenon [1]. In healthy individuals it is normal and temporary whereas disease-related fatigue is dysfunctional, longer-lasting or chronic and persists despite adequate periods of rest and sleep [2-4]. Fatigue is a significant component of many chronic inflammatory conditions [1].

Historically, fatigue associated with ankylosing spondylitis (AS) has received little attention. [5]. However, more recent studies have now demonstrated that over half of people with AS experience this debilitating phenomenon [5-8], with fatigue now accepted as a core symptom of the condition.

The fatigue experienced by people with AS is reported to be related to disease activity [5,9-12], poorer functional ability [5,10,12,13], pain [5,13,14] and stiffness [5,10,13] mental health problems [9,11,13] such as depression [11], lower global well-being [10,13], impaired working [13] and enthesitis [10]. Understandably, due to the symptoms experienced, an additional factor associated with AS-related fatigue is sleep. Studies report that more than half of individuals with AS have sleep disorders related to fatigue [7,11,14].

Amitriptyline medication is often prescribed for fatigue management in AS [5], and current guidelines from the National Institute for Health and Care Excellence (NICE) recommend utilising psychological interventions to help in the management of inflammatory arthritis [15].

The aim of this study is to explore from the patients’ perspective (i) the effect of fatigue in AS with a focus on (ii) ways of self-managing fatigue as part of their everyday lives, and (iii) to identify potential interventions for future research to help combat fatigue in AS.

## Method

This study builds on our existing Population-based Ankylosing Spondylitis (PAS) cohort study that currently involves 500+ participants with AS. The protocol for the development of the cohort has previously been published [16]. Briefly 500+ patients with AS residing in Wales, UK were recruited between 2008 and 2011 and were sent questionnaires every 3 months to examine disease activity (including pain and fatigue), function, medication, and quality of life. Respondents were asked specific questions about flares, exercise, work, health care costs, and coping with AS. Two open-ended questions (essay type) were included in the questionnaire exploring the individual’s experience of fatigue and personal management strategies. Following on from this quantitative study [17] which identified that management strategies for fatigue in AS should focus on pain management including psychological interventions, we wanted to conduct focus groups with people with AS to further explore potential management strategies for fatigue in AS, to help inform our research agenda for potential future research activity stemming from the patients’ perspective.

Focus groups are advantageous for data collection, as group interaction enhances the depth of conversation due to stimulation of thoughts from other group members, and responses can be shared and compared [18].

### Ethics

The PAS study received ethical approval from the London Multi-centre Research Ethics committee and participants gave written consent in accordance to the Declaration of Helsinki. Approval for the focus groups was obtained from the same Ethics committee via a separate substantial amendment.

### Participants

Participants (n = 102) in the PAS cohort study resident in the catchment area for the Abertawe BroMorgannwg University Health Board (ABM UHB) Wales, UK were sent an invitation pack, which included an invitation letter, information sheet, focus group schedule expression of interest (EOI) form, and consent form. The locality of ABM UHB was chosen as Swansea is Wales’ second largest city and its population diverse. Latest figures shown that Swansea has a population of 229,100 [19] and in terms of employment, Swansea has the second most deprived and the least deprived areas in Wales [20].

### Focus groups

Two focus groups were conducted in October 2011 in local venues (hotels) in the locality of ABM UHB. The focus groups lasted approximately 1 hour 45 minutes. One researcher (HD) moderated the focus group discussion, and one project assistant (EI) provided technical support and participant observation (note taking of non-verbal communication).

In order to assess if participants in the focus groups were representative of other people with AS we compared characteristics (age, gender, disease duration and fatigue levels) of participants who attended a focus group from ABM UHB (n = 14) with (i) all PAS participants (n = 533), and (ii) PAS participants from ABM UHB who declined to participate in the focus group (n = 18).

### Focus group schedule

The focus group schedule was developed using broad topic areas which complemented and lead on from two original open-ended questions in a previous PAS study questionnaire on fatigue [17]. Consideration was also given to existing literature, and current clinical practice in relation to managing fatigue in AS. We wanted to specifically explore views and experiences of two diverse strategies for managing fatigue; medication and psychological interventions. Representing these avenues for discussion we choose amitriptyline (medication), and Mindfulness-Based Stress Reduction (MBSR) (psychological intervention) respectively. The rationale being amitriptyline is frequently prescribed for people with AS and has been shown to improve fatigue [5], and the NICE guidelines (2009) recommend that people with inflammatory arthritis should be offered access to psychological interventions to help manage their condition [15]. In particular, MBSR has been found to be beneficial for chronic pain [21] which is key for the management of fatigue [17]. Participants in this study were encouraged to discuss other treatments for fatigue which fall between the two diverse strategies chosen, thus providing an open forum in which to explore other management strategies of interest to help inform future research. The focus group schedule was flexible to encourage a broad spectrum of participant’s views. All participants were sent the focus group schedule at least 3 weeks in advance.

### Data management and data analysis

Each focus group session encompassed interaction and responses between participants and moderator which were recorded and transcribed verbatim in Microsoft Word. Each verbatim transcription then served as a primary text document. Using a qualitative thematic analyses [22] each primary document was read several times by (HD and SB) to gain theoretical sensitivity which emphasises the participants’ frame of mind [23]. A researcher (HD) used open coding for each quote, conversation or paragraph in an attempt to encapsulate participants’ meaning. Another researcher (SB) checked the codes for accuracy and consistency with no discrepant codes identified. Related and reoccurring codes were grouped together to form main themes and specific quotations from the primary documents were highlighted to illustrate the salient themes.

Personal data generated from this study was anonymised using unique identification numbers and all paper data was securely stored in locked cupboards and electronic data in password protected files on a secure university server.

## Results

Of 102 invitations sent, 45 (46%) potential participants returned the EOI form with 24/45 (53%) expressing interest in participating in a focus group. 18/45 (40%) were not interested (declined) and 3/45 (7%) returned a blank EOI form. Reasons for not participating in this study included, ill-health, being a full-time carer and not wanting to join a group discussion. 14/24 (58%) who expressed an interest, participated in one of the two focus groups. 10/24 (42%) were not available on the dates selected for the focus groups. All participants (n = 14, 7 male) had a clinical diagnosis of AS with an average disease duration (based on date of diagnosis) of 29 years (range 8–46 years). Participants consisted of 3 males/4 females (group 1) and 4 males/3 females (group 2), aged between 35 and 73 years (mean age 53 years). All participants were Caucasian. One participant (1/14) was on a tumor necrosis factor (TNF) blocker.

The characteristics of the participants who attended the focus groups (n = 14) were comparable with those who declined (n = 18) and comparable to the population from which they were selected (all PAS participants (n = 533)) for age: mean age was 50, 49 and 53 years, respectively. Disease duration: was slightly longer (29 years) in the focus group participants compared with 18 years and 22 years, for declined and population, respectively. However, the percentage of females appeared higher for those attending the focus group 50% (7/14) compared to the population, 25% (134/533). In addition, the fatigue levels appeared higher in the focus group participants 72% (10/14) compared to those who declined to participate 50% (9/18).

This study identified 3 main themes; [1] Pervasive fatigue; [2] current limitations (of self-management); and [3] a new direction (for future interventions).

(1) Pervasive fatigue

Participants reported many ‘life’ influences which exacerbated or contributed to feelings of fatigue including; age, lack of sleep, night time pain, low-mood, depression, lack of concentration, side effects from medication, work commitments and the unpredictability of the condition. One participant felt it was difficult to distinguish *‘age-related’* symptoms from fatigue in AS *“I nod off in the chair, but that’s not fatigue, that is just getting old and getting tired”.* Other participants make a clear distinction that fatigue is exacerbated by lack of sleep caused by pain *“I can’t remember ever having a good night’s sleep. I wake up constantly every couple of hours through pain, I never sleep through, so I am constantly tired”,* which in turn causes physical symptoms such as “*a burning sensation with your eyes when you are really tired”.*

The majority of participants expressed that side-effects from analgesic medication also contributed to feelings of fatigue with many participants expressing concern that their medication was adding to their fatigue *“I always put it [fatigue] down to my pain and then I wasn’t sure if it was the medication…other members of my family also take high doses of co-codamol and it sends them to sleep…”* Also, fatigue is unpredictable *“you can get this tiredness for no reason. It just comes on you…”* whereas another participant implied *“I never found that I was tired generally, only after a flare up.”*

Work commitments were on one hand viewed as a motivator for life, one participant said “*it* [job] *keeps me going”,* but alternatively another participant who finished work due to fatigue in AS stated *“I have finished work now so I am managing much better, but when I was working I found it very hard, you could just cry or you felt physically sick because you felt so tired.”* In agreement one participant stated “*In the end I just gave up work…it made it easier physically not having to go in, but I still find I am having periods of mood swings and depression…the longer it* [sleep problems] *goes on then the more mood swings kick in.”* Another participant found it *“hard to concentrate in work because you felt so tired.”*

There was a general consensus over the uncertainty of whether the symptoms of AS (such as pain, impaired sleep) caused fatigue or if fatigue was a symptom of AS. One participant questioned *“Has the humira* [adalimumab] *helped me with the fatigue or has it helped with my sleeping pattern which has indirectly helped with my fatigue?”*.

The next theme highlights self-management interventions used by participants including experiences and challenges.

(2) Current limitations (of self-management)

Participants were encouraged in the focus group to discuss what interventions or treatments they had tried, or wanted to try with regard to self-managing their fatigue. Responses ranged from; having a positive attitude *“just keep on going” and “trying to see the glass half full not half empty”*, self-medication *“I will take more co-codamol”*, keeping active *“every day I walk for a good hour with the dogs, I swim and I keep active”*, and being a member of a gym. Hydrotherapy was a popular activity, but finding a warm pool and the cost involved were viewed as problematic “*it would be lovely to swim in a warm pool but the prices these places are charging.”* One participant stated they felt they *“had to exercise that was the leveller…..if I don’t do it* [exercise] *I am in real trouble.”* Other participants reported sleeping for short periods of time *“a 10 minute power nap and I am fine again”.*

Some participants implied they tried not to rely on medication for managing their AS due to the many side-effects experienced. The following quote illustrates this situation: *“I was taking indomethacin for the pain but it caused the bowel to flare up and I was hospitalised with that….so I try and manage without drugs if I can…but of course you have to if the pain is very bad.”*

As part of the focus group participants were shown a 3 minute video clip on amitriptyline medication [24] (which can be prescribed before bedtime for fatigue and pain in chronic musculoskeletal conditions), including how it works, dosage and its advantages for managing chronic nerve pain. Four out of 14 participants had used with this type of medication previously. Ten participants had no previous knowledge *“I have never heard of it”*. Participants who had taken amitriptyline complained of side-effects “…*I took it for a couple of months…..it just made me feel like a zombie…I felt worse”*. Another participant experienced a dry mouth and feeling drowsy in the morning. One participant who had taken amitriptyline for over 2 years reported no side-effects but implied the drug made no difference to her symptoms *“I honestly don’t see any difference really, I mean I was wandering around at 3 o’clock this morning…I couldn’t sleep…”.* The fourth participant with experience of amitriptyline stated she was wary of taking an antidepressant *“It frightened me a bit when they [rheumatologist] mentioned antidepressant….I thought that it might be addictive but I didn’t know so I took it for a week then I stopped just in case I got addicted to it.”*

A few participants had tried alternative or complementary interventions for managing fatigue. One participant tried meditation guided by an occupational health advisor which was *“lovely”* but not very useful when experiencing a flare *“I didn’t find that really very good for when you are having a bad time which is really when I thought it would be.”* Acupuncture, TENS machines and heat wraps were described as *“pleasant”,* but provided only *“temporary”* pain relief. One participant suggested that breathing exercises *“worked if you had real acute pain and you tried to focus on your breathing”.* Yoga and Pilates activity seemed to be avoided due to fear of *“over doing it”* especially during a *“flare-up”.*

(3) A new direction (for future interventions)

Participants were also shown a 3 minutes video clip on MBSR [25], a psychological intervention which has been shown to have physical and psychological benefits in numerous health conditions [26] and was originally developed for managing chronic pain [27]. None of the participants had heard of MBSR and only one had tried a psychological intervention (cognitive behaviour therapy – CBT) for depression and anxiety, rather than management of fatigue in AS.

The majority of participants in this study were open to trying MBSR and many requested more information about available courses. The following quotes illustrate this: One participant stated “*You never know with these things [psychological interventions] sometimes they work and sometimes they don’t but it is well worth trying isn’t it really.”* Another participant commented, *“I think it* [MBSR] *is something that I would like to try… I would give it a go”.* Several participants appeared very enthusiastic expressing *“I would like some more information about this”* and *“If there was a chance for me to go on a course I would go*” and *“I would be very happy in experiencing that sort of technique.”* It was also suggested that MBSR should be available to significant others or carers of the person with AS, with one participant saying *“I think my wife would benefit from it and her stress levels…”*

The group discussion evolved towards the different delivery modes of MBSR which is traditionally delivered in a weekly group session of 2.5 hours over 8 consecutive weeks. Other potential modes of deliver explored included on-line courses and distance delivery over the telephone with a similar time delivery (over 8 weeks). Most participants in this study expressed a preference for the traditional group structure rather than on-line or distance delivery.

However, reasons were put forward to advocate the MBSR delivery method to fit the needs of the individual: *“When I was working I would have found it difficult to commit to 1 day a week for 8 weeks, so the distance delivery might be better.”* And another participant commented *“There will always be people that don’t want to talk about it to another person”* and *“there would have to be something that would suit all people not just one thing* (one delivery mode)”.

When probed for future research ideas for fatigue in AS it became apparent that most participants wanted more *‘alternative’* or *‘psychological therapy’* interventions funded rather than focusing on more drug trials and research into orthodox medication. The following quotes illustrate this view: *“I think there is a lot of research when it comes to drugs in AS and there is not a lot of research which it comes to alternative therapies”* and *“I think the answer is to research different things”*

## Discussion

To our knowledge this is the first qualitative study exploring management strategies for fatigue from the patient perspective. Both focus groups followed the structure outlined in the schedule with participants first discussing the perception and lived-experience of fatigue in AS. The participants in this study appear to experience fatigue on a daily basis but they remained unsure as to whether the symptoms of fatigue was a component of the condition itself, or whether the fatigue experienced was a result of having disturbed sleep due to chronic pain. The general consensus in the literature is that fatigue is a feature of inflammatory conditions, including AS [1,28,29]. Other studies [11,17,30,31], indicate that pain is the most powerful predictor of fatigue. More recently an editorial on fatigue in rheumatoid arthritis concluded that the mediation of fatigue relates more to psychological and physical factors such as anxiety, depressive symptoms, pain disability and sleep disturbances and much less to the consequences of the disease process [32]. However, a conceptual model encompassing factors related to disease process, cognitive/behavioural and personal life issues has been proposed for rheumatoid arthritis [28].

Regardless of the origin, fatigue is an issue for many patients with AS [6-8,30,33,34], with self-management strategies being advocated for optimal functioning and daily living [35]. Our study highlights the universal, yet individual and subjective experience of fatigue in AS. Participants managed their pain by self medicating which often proved (i) limited or ineffective for many participants, while (ii) others experienced undesirable side-effects and others (iii) feared over reliance and addiction. The medication consequences often exacerbated feelings of fatigue and loss of control.

This study also explored how participants currently managed fatigue and ideas for future strategies. Self-management of a disease like chronic arthritis has many facets. The Institute of Medicine in the US defines it as “relating to the tasks that an individual must undertake to live well with one or more chronic conditions [36]. These tasks include gaining confidence to deal with medical management, role management and emotional management”.

Our participants mainly relied on self-medication, and a balance of exercise and short periods of sleep *‘nodding off’* to help combat the feelings of fatigue. The latter option was only viable to those participants who had either retired or were not working. Some participants gave up employment commitments due to their AS and stated to be managing their condition and fatigue better since leaving work, again indicating the role of behavioural and personal factors on their fatigue.

All participants in this study were *‘happy’* and *‘willing’* to try different interventions to help manage fatigue in AS including psychological interventions such as MBSR, and would prefer to integrate alternative and psychological interventions with traditional medication rather than medication on its own. This approach would be consistent with the multi-factorial causative model for fatigue in inflammatory arthritis [28]. Interestingly, participants also advocated MBSR for their carers or significant others to help them deal with the stress of living with someone with AS. While group-based MBSR was the preferred method of delivery by participants in this study, it was acknowledged that different delivery modes of MBSR (on-line, distance delivery) were necessary to reach people with work or family commitments, and those who do not like participating in a group environment.

By comparing characteristics of AS participants in our focus groups with the characteristics of other people with AS we can suggest that males with AS and with low levels of fatigue are less likely to attend group work, hence psychological interventions aimed at this population should include a non-group delivery option i.e. individual distance delivery. However, people with AS (including males) and high levels of fatigue appear to advocate group work.

A limitation of the study is that our two focus groups might not be representative of the opinions and experiences of all people with fatigue associated with AS. Participants’ views on preference for MBSR being delivered in a group may be potentially biased as people who attended focus groups are more likely to be advocates of group work. However this qualitative exploratory study (which is concerned with an in-depth understanding of a topic rather than the generalizability of findings) is the first study taken from a patient perspective in this topic area and our preliminary findings provide insight into potential and future management strategies for fatigue in AS.

In addition, we do not know if participants in this study discussed fatigue with their healthcare providers, but we do know that participants had unresolved fatigue issues whether this had previously been discussed or not.

## Conclusions

In conclusion, there is currently no specific agreed treatment for the management of fatigue in AS. In the UK, NICE guidelines (2009) for rheumatoid arthritis it recommends offering access to psychological interventions to help manage the condition [37], which is also applicable to AS and other forms of inflammatory arthritis. Quantitative research [17] has shown that fatigue is multidimensional, and this qualitative study from the patients’ perspective has shown support for the use of psychological interventions for the management of fatigue in AS. Following the findings from our study, our future research agenda will endeavour to include further studies examining psychological interventions including MBSR in AS.

The way forward is for the Rheumatology community to recognise the full impact of fatigue in AS, and embrace and refer patients to psychological therapies, including MBSR, which have increasing evidence for their role in fatigue as well as being acceptable to patients who may already be taking several medications for their underlying condition.

## Competing interests

The authors declare they have no competing interests.

## Authors’ contributions

Conceived and designed the focus group: HD, SB, SS. Moderated and facilitated the focus group: HD, EI. Analysed the data: HD, SB. Wrote the paper: HD, SB, MD, EI, RC SS. All authors read and approved the final manuscript.

## Pre-publication history

The pre-publication history for this paper can be accessed here:

http://www.biomedcentral.com/1471-2474/14/163/prepub
